# Nanotechnology-driven nanoemulsion gel for enhanced transdermal delivery of *Sophora alopecuroides L.* empyreumatic oil: formulation optimization, and anti-biofilm efficacy

**DOI:** 10.3389/fbioe.2025.1586924

**Published:** 2025-04-25

**Authors:** Xiuli Cheng, Xiangyu Zhou, Wenping Wang, Jing Chen, Yikun Cao, Jia Wen, Jin Hu

**Affiliations:** ^1^ Department of Pharmacy, People’s Hospital of Ningxia Hui Autonomous Region, Yinchuan, China; ^2^ Preperation Center, General Hospital of Ningxia Medical University, Yinchuan, China; ^3^ Center of Neurological Disorders, Shizuishan First People’s Hospital, Shizuishan, China; ^4^ Yunnan University of Traditional Chinese Medicine, Yunnan University of Chinese Medicine, Kunming, China; ^5^ General Medicine Department, General Hospital of Ningxia Medical University, Yinchuan, China

**Keywords:** *Sophora alopecuroide L.*, empyreumatic oil, nanoemulsion gel, skin permeation, irritation, anti-biofilm

## Abstract

*Sophora alopecuroides L.* empyreumatic oil (SoA oil) exhibits therapeutic potential for psoriasis and eczema but suffers from poor skin permeability and formulation challenges. To overcome these limitations, a nanoemulsion (NE) gel was developed. The NE was optimized using pseudo-ternary phase diagrams and characterized for droplet size, polydispersity index (PDI), zeta potential, and rheological properties. Skin permeability and retention were assessed *in vitro* using Franz diffusion cells, with oxymatrine quantified by HPLC. *In vivo* skin irritation was tested on rabbit dorsal skin, and anti-biofilm activity was evaluated against *Staphylococcus aureus* (*S*. *aureus*) and methicillin-resistant *S. aureus* (MRSA). A final concentration of 5% SoA oil in the NE formulation was used for subsequent studies. The optimized SoA oil NE (the NE) had a mean droplet size of 53.27 nm, PDI of 0.236, and zeta potential of −38.13 mV. Adding 2% carbomer 940 (CP940) to the gel enhanced viscoelasticity. The NE showed superior skin permeability and higher cutaneous retention of oxymatrine. SoA oil caused moderate irritation to the skin of rabbits, while the other two formulations did not. The NE demonstrated enhanced biofilm inhibition against *S. aureus* at 0.09766 mg/mL, with an 8.9% rate surpassing SoA oil (2.0%) and SoA oil NE gel (the gel, 4.0%). At 12.50 mg/mL, the NE and the gel achieved slightly higher inhibition rates (81.7% and 82.1%, respectively) than SoA oil (78.3%). Notably, the NE showed significantly greater anti-biofilm effects against MRSA within the concentration range from 0.09766 to 3.12 mg/mL (*P* < 0.001). In mature biofilm clearance against *S. aureus*, the NE demonstrated a clearance rate of 4.9% at 0.09766 mg/mL, while SoA oil and the NE gel achieved clearance rates of 2.3% and 0.8%, respectively. At a higher concentration of 12.50 mg/mL, the clearance rate for the NE increased to 38.1%, significantly outperforming SoA oil (29.1%) and the NE gel (36.4%). Against MRSA, the NE and the gel displayed significantly improved clearance at 12.50 mg/mL (42.7% and 43.9%, respectively) compared to SoA oil (31.9%) (*P* < 0.0001). These findings highlight the potential of nanotechnology-driven delivery systems to improve the clinical application of herbal extracts for treating biofilm-associated dermatological infections.

## 1 Introduction

**FIGURE 1:**
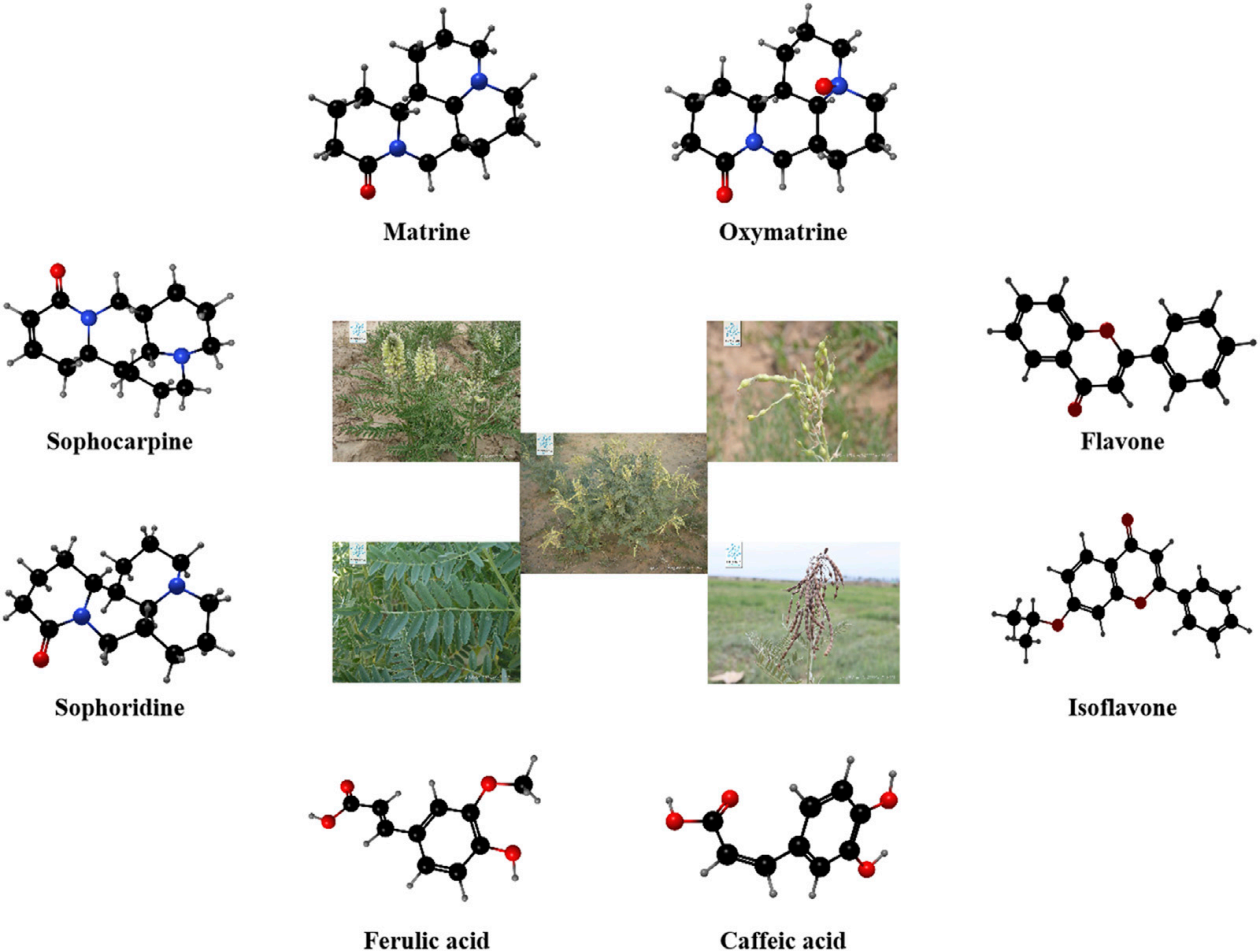
The appearance of *Sophora alopecuroides L.* (Images quoted from Plant Photo Bank of China) and the corresponding three-dimensional structural formula of the part metobolites it contains.

*Sophora alopecuroides L.* (SoA), a member of the Fabaceae family, is a leguminous herbaceous perennial widely distributed in Northwest China (Rong et al., 2020; Rong et al., 2024). This plant is rich in phytochemicals, with hundreds of metabolites identified, including alkaloids (Cao et al., 2024), flavonoids (Zhu et al., 2023), steroids, and polysaccharides (Guo et al., 2016) (Figure 1). These active metabolites of SoA exhibit a wide range of medicinal properties, such as anti-tumor (Li et al., 2021), antiviral, anti-inflammatory (Luo et al., 2024), antibacterial (Wan et al., 2015), analgesic (Huang and Xu, 2016), and neuroprotective activities (Sun et al., 2023).

SoA empyreumatic oil (SoA oil) is prepared through vacuum distilled. It has been traditionally used in folk medicine for treating eczema (Yuan et al., 2013a), psoriasis vulgaris (Chen and Li, 2010), and neurodermatitis (Yuan et al., 2013b). However, SoA oil has several limitations, including poor skin permeation, and inadequate retention in the skin. Additionally, its dark color and unpleasant odor, along with its tendency to adhere to wounds and surrounding skin upon topical application, can cause discomfort for patients, thereby limiting its clinical use.

Nanoemulsion (NE) has emerged as a promising approach for transdermal drug delivery (Roy et al., 2022; Souto et al., 2022). The components of NE can act as permeation enhancers, potentially reducing the diffusional barrier posed by the stratum corneum and increasing the rate of drug permeation through the skin (Nastiti et al., 2017). Furthermore, the hydration effect of NE on the stratum corneum can modulate the permeability of drug formulations (Zhu et al., 2020).

This study aims to develop a NE gel of SoA oil to enhance its transdermal delivery, and improve antibacterial activity against biofilm-forming pathogens. By optimizing SoA oil NE (the NE) formulation and evaluating its physicochemical properties, skin permeability and irritation, this research seeks to address the limitations of SoA oil and provide a scientific basis for its clinical application as a potential therapeutic agent for biofilm-associated skin infections.

## 2 Materials and methods

### 2.1 Materials

The SoA oil was sourced from Xinjiang Hope Pharmaceutical Co., LTD. (Xinjiang, China). Methanol was procured from Sigma-Aldrich (St. Louis, MO, United States), while Tween 80, glycerol, cremopher EL, PEG 400 and oxymatrine were obtained from MCE^®^ (California, United States). Carbomer 940 was acquired from Aladdin (Shanghai, China), and sodium carboxymethyl cellulose (CMC-Na) as well as hydroxypropyl cellulose (HPMC) were purchased from Yunhong Excipients (Shanghai, China) and Chineway (Shanghai, China), respectively.

### 2.2 Animals and bacteria

The skin of Kunming rats were used for drug permeability test, and rabbits were used for drug skin irritation assay. Ethics approval was obtained from Medical Research Ethics Review Committee, General Hospital of Ningxia Medical University before the study began (KYLL-2021-707). *Staphylococcus aureus* (*S. aureus*, ATCC 6538) and methicillin-resistant *S. aureus* (MRSA, ATCC 43300) were obtained from the Guangdong Microbial Culture Collection Center.

### 2.3 Content analysis of oxymatrine assay

Studies have shown that oxymatrine constitutes up to 12% of the total alkaloids in *S. alopecuroides L* (Chen et al., 2016), therefore, was chosen as the indicator for subsequent quantitative analysis. The concentration of oxymatrine was determined using an optimized High-Performance Liquid Chromatography (HPLC) method, based on previous reports with some modifications (Bi et al., 2010). The analysis was performed on an EClassical 3100 Series HPLC system (Dalian Elite Analytical Instruments Co., Ltd., China) equipped with a SupersilODS2 C18 column (4.6 mm × 200 mm×5 μm). The mobile phase consisted of a mixture of methanol and an aqueous solution of triethylamine (0.03%) in a ratio of 25:75 (v/v), with a flow rate set at 1.0 mL/min. Detection was performed at a wavelength of 210 nm, with the system temperature maintained at 25°C. The sample injection volume was 10 μL. Under these conditions, the retention time for oxymatrine was approximately 6.0 min, and no interference from other metabolites was observed.

### 2.4 Constuction of pseudo-ternary phase diagram

Pseudo-ternary phase diagrams were carefully constructed using the aqueous titration method. Surfactants (Tween 80, Cremophor EL, or Labrasol) and co-surfactants (glycerol, ethanol, or PEG400) were combined in weight ratios of 3:1, 2:1, 1:1, 1:2, and 1:3 (w/w) to prepare the surfactant mixture (Smix). For each phase diagram, SoA oil was mixed thoroughly with Smix in various weight ratios ranging from 1:9 to 9:1. In each vial containing a specific oil-to-Smix ratio, water was added incrementally under gentle stirring. The physical state of the system was then visually assessed and documented. The proportions of different phases at each stage were calculated and plotted on a pseudo-ternary phase diagram using Origin software (version 9.0).

### 2.5 Characterization of NE

The mean droplet size, polydispersity index (PDI), and zeta potential of optimized NE formulations was assessed by laser particle analyzer (90Plus PALS, Brookhaven, United States). The measurements were performed at 25°C, with a detection angle of 90°. The morphological characteristics of the NE were examined using transmission electron microscopy (TEM, H-7650, Hitachi, Japan). We prepared samples by placing a drop of the diluted NE on a carbon-coated copper grid and allowing it to dry. The TEM images were captured at an accelerating voltage of 100 kV and analyzed using Hitachi imaging software to assess the shape and size distribution of the droplets.

### 2.6 Screening and rheological studies of SoA oil NE gel

To further improve the stability and drug retention of the NE, individual additions of 2% sodium carboxymethyl cellulose (CMC-Na), hydroxypropyl cellulose (HPMC), and carbomer 940 (CP940) were introduced into NE formulations. Subsequent to the gel’s complete swelling, rheological measurements were carried out with a controlled stress rheometer (MCR 302, Anton Paar, China), equipped with a PP25 measuring system and a parallel plate geometry. The top plate (25 mm diameter) applied shear, and the bottom plate maintained the temperature to an accuracy of 0.01°C. The storage modulus (G′) and loss modulus (G″) were continuously observed in relation to temperature changes, utilizing two distinct sample thicknesses that were determined by the gap between parallel plates.

### 2.7 *In vitro* skin permeation and drug retention study

Franz diffusion cell method was employed to analysis the skin permeation and drug retention. In brief, dorsal skin samples were harvested from Kunming rats, with subcutaneous fat and connective tissue meticulously removed. The skin was then mounted between the donor and receptor chambers, positioning the stratum corneum side upwards. Subsequently, 1.0 mL of SoA oil, the NE and SoA oil NE gel (the gel) were applied to the skin’s surface, encompassing an effective diffusion area of 2.89 cm^2^, with phosphate buffer saline (PBS, pH 6.8) used as the receptor medium. This medium was constantly stirred at 300 rpm using a magnetic stirrer and maintained at a temperature of 37°C ± 0.5°C throughout the experiment. At predetermined intervals-0.5, 1, 2, 4, 6, 8, 10, and 12 h-the receptor phase was carefully withdrawn and promptly replaced with an equivalent volume of fresh medium. The concentration of oxymatrine in each sample were quantitatively determined via HPLC method.

After completing the *in vitro* skin permeation experiment, the rat skin, encompassing the effective transdermal area, was removed. The surface remnants of the receiving solution and sample solutions were gently eliminated using a cotton swab dipped in a 0.9% sodium chloride solution. The treated rat skin was then diced into small segments and placed into a centrifuge tube. A volume of 5 mL of methanol was added, subsequently subjected to ultrasonication for a duration of 15 min, then centrifuged at 1,200 rpm for 10 min. The supernatant was then filtered through a 0.22 μm filter, and the content of oxymatrine was measured using HPLC method. Skin retention of the drug was computed employing Formula 1 (Zou et al., 2023).

$$Q_{s}=V’C/A$$ (1)

*Q*_*S*_ represents the skin retention content, *V′* represents the total volume of extracting rat skin fluid, *C* represents the total concentration of drugs in the rat skin extract, and *A* represents the diffusion and permeation area (2.89 cm^2^).

### 2.8 Skin irritation test

This study was conducted to assess the irritant potential of the developed formulations following topical application. The hair on the dorsal side (2 cm × 3 cm) of six rabbits were removed using hair removal cream, taking care not to damage the skin. On the dorsal skin of each rabbit, five different areas were treated with distinct formulations. The saline group was treated with saline, the control group was treated with NE gel without SoA oil, the treatment groups received applications of SoA oil, the NE, and the gel, respectively, once daily for seven consecutive days. After the 7-day period, the application sites were gently cleansed with warm water. Erythema and edema were then evaluated at 1, 24, 48, and 72 h post-application. Skin was carefully examined for signs of edema and erythema. Skin lesions were scored as follows: no erythema/edema, 0 points; slight erythema/edema, one point; well-defined erythema/edema, two points; moderate erythema/edema, three points; and scar formation, four points (Hussain et al., 2016).

### 2.9 Anti-biofilm activity assay

#### 2.9.1 Bacteria strains and culturing condition

The bacterial strains were revived in Luria-Bertani (LB) broth (Mille™, 12795084, United States) and incubated at 37°C for 24 h, following a previously established protocol with some modified (Liu et al., 2021). We prepared the standardized inocula by diluting the cultures to a concentration of 1.5 × 10^6^ colony-forming units (CFU/mL) in LB broth.

#### 2.9.2 Biofilm inhibition assay

*S. aureus* and MRSA strains were activated by culturing in LB medium at 37°C for 24 h. A single colony from each strain was inoculated into 100 mL fresh LB medium and incubated for 18 h at 37°C. Bacterial suspensions were standardized to 1.5 × 10^6^ CFU/mL using McFarland turbidity standards. Aliquots (100 μL) of LB medium containing NE or NE gel (concentrations: 12.50–0.10 mg/mL) were dispensed into a sterile 96-well plate. Ciprofloxacin (20 μL) and untreated bacterial suspensions served as negative and positive controls, respectively. Bacterial suspensions (100 μL) were added to each well, followed by incubation at 37°C for 24 h. Biofilm biomass was quantified via crystal violet staining, and inhibition rates were calculated using Formula 2:

$$Inhibitionrate=\left(\frac{ODpositive\;control-ODdrug}{ODpositivecontrol-ODnegativecontrol}\right)\times 100$$ (2)

#### 2.9.3 Mature biofilm clearance assay

Log-phase bacterial suspensions (1 × 10^6^ CFU/mL) in LB medium supplemented with 1% glucose were aliquoted (100 μL) into 96-well plates. Plates were incubated statically at 37°C for 72 h, with medium replacement every 24 h to maintain nutrient availability. Post-incubation, biofilms were gently washed thrice with sterile PBS. Test compounds (NE or NE gel, 100 μL; concentrations: 12.50–0.10 mg/mL), ciprofloxacin (negative control), and untreated suspensions (positive control) were added. After 24 h static incubation at 37°C, residual biofilm biomass was stained with CV. Biofilm clearance efficiency was determined using Formula 3:

$$Clearance\;rate=\left(1-\frac{OD\;drug}{OD\;positive\;control}\right)\times 100$$ (3)

### 2.10 Statistical analysis

Statistical analysis was conducted using GraphPad Prism 9.5. One-way analysis of variance (ANOVA) was performed, followed by Dunnett’s or Tukey’s multiple comparisons test to evaluate differences among the various treatment groups. Significant results are detailed in the figure captions.

## 3 Results and discussion

### 3.1 Pseudo-ternary phase diagram

**FIGURE 2:**
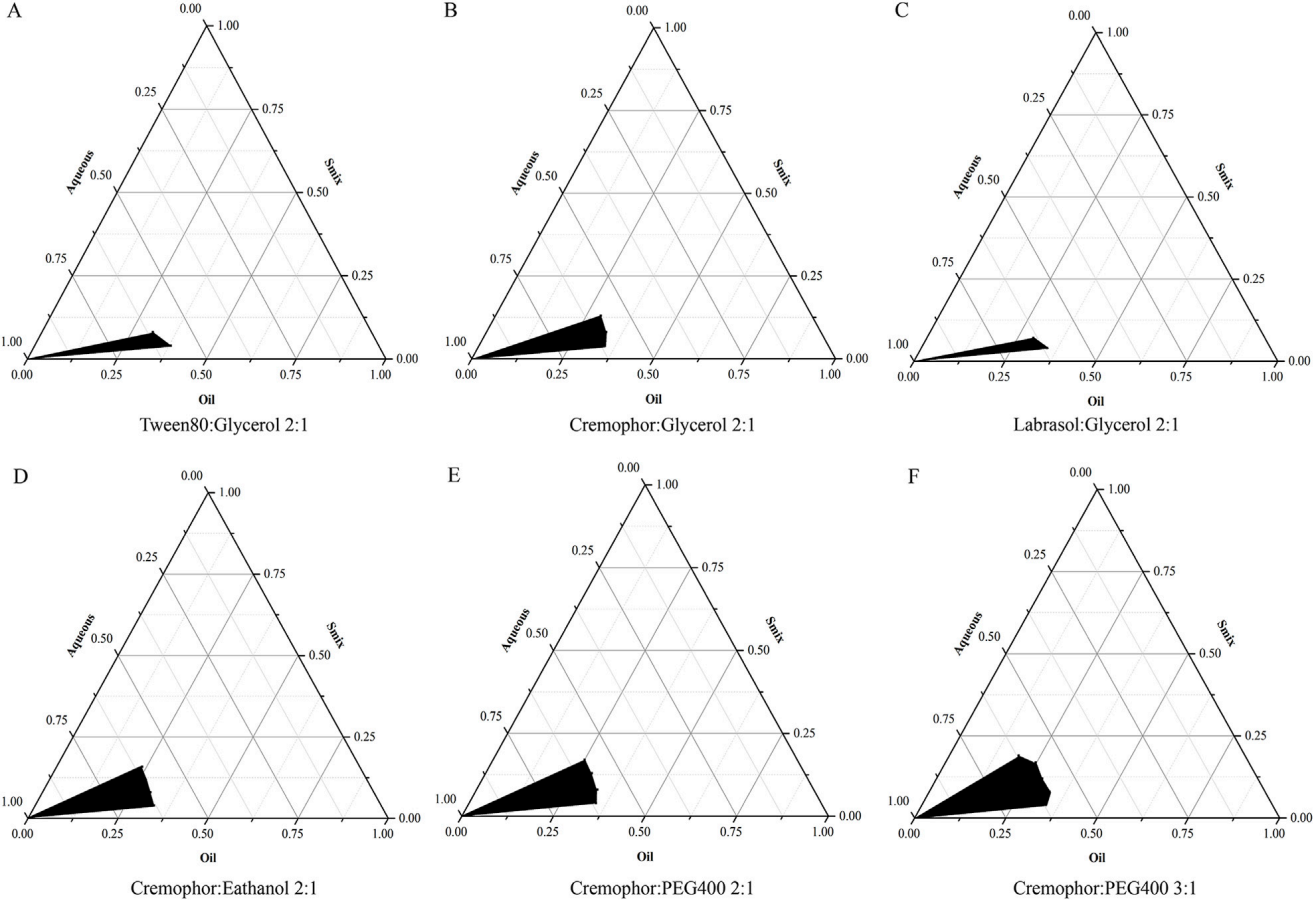
Pseudo-ternary phase diagrams for NE systems, highlighting the stable oil-in-water NE region in black. The components are as follows: **(A)** is a Tween80 and glycerol mixture, **(B)** is cremophor and glycerol, **(C)** is labrasol and glycerol, **(D)** is cremophor and ethanol, **(E)** is cremophor and PEG400, each with a Km of 2:1, and **(F)** is cremophor and PEG400 at a 3:1 ratio.

As shown in Figure 2, at a Km value of 2:1, with glycerin serving as the co-surfactant, the largest NE region was achieved utilizing cremophor EL as the surfactant, exhibiting an area of 5.87% (Figure 2B). The NE areas obtained with Tween80 and labrasol were 4.17% and 3.75%, respectively (Figures 2A, C), leading to the selection of cremophor EL as the optimal surfactant. Maintaining the Km value at 2:1 and employing cremophor EL as the surfactant, the largest NE region was observed when PEG400 was employed as the co-surfactant, reaching an area of 6.22% (Figure 2E), and using ethanol as the co-surfactant resulted in an NE area of 3.55% (Figure 2D), thereby PEG 400 was selected as the most efficacious co-surfactant. In formulations where cremophor EL acted as the surfactant paired with PEG 400 as the co-surfactant, no successful NE formation was noted at Km ratios of 1:1, 1:2, or 1:3. However, a significant improvement was observed at a Km ratio of 3:1, with the NE area expanding to 8.96% (Figure 2F), surpassing that of the 2:1 Km ratio. Consequently, the optimal formulation strategy for preparing the NE entails the use of cremophor EL as the surfactant, PEG 400 as the co-surfactant, and a Km ratio set at 3:1. The concentration of SoA oil in the optimal NE formulation has been adjusted to 5% for subsequent research.

### 3.2 Characterization of the NE

**FIGURE 3:**
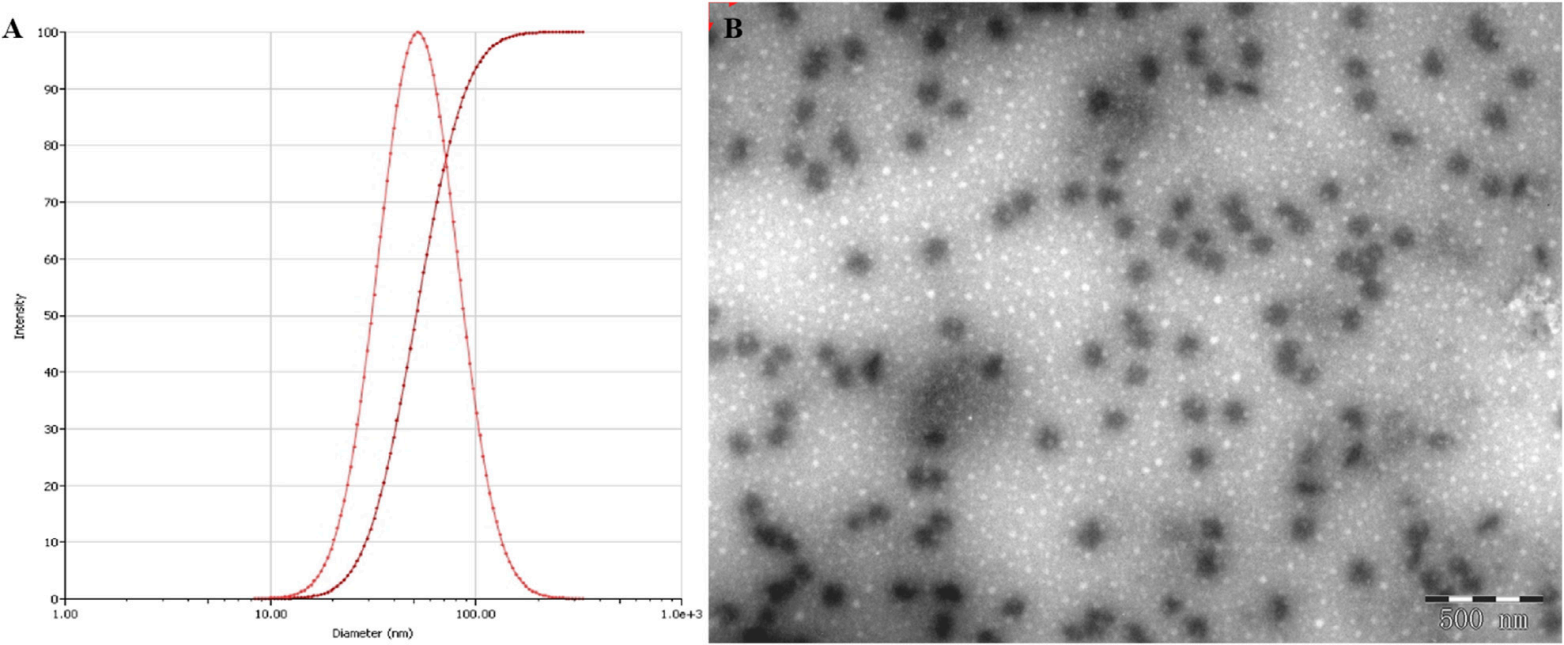
Droplet size, distribution **(A)** and TEM images (size bar = 500 nm) **(B)** of NE.

The NE displayed an average droplet size of 53.27 nm (Figure 3A), a PDI value of 0.236, which were further confirmed by TEM observation (Figure 3B), which indicate that NE possessed a small particle size with a low PDI value, the zeta potential of NE was −38.1 mV.

### 3.3 Rheological properties of the gel formulations

**FIGURE 4:**
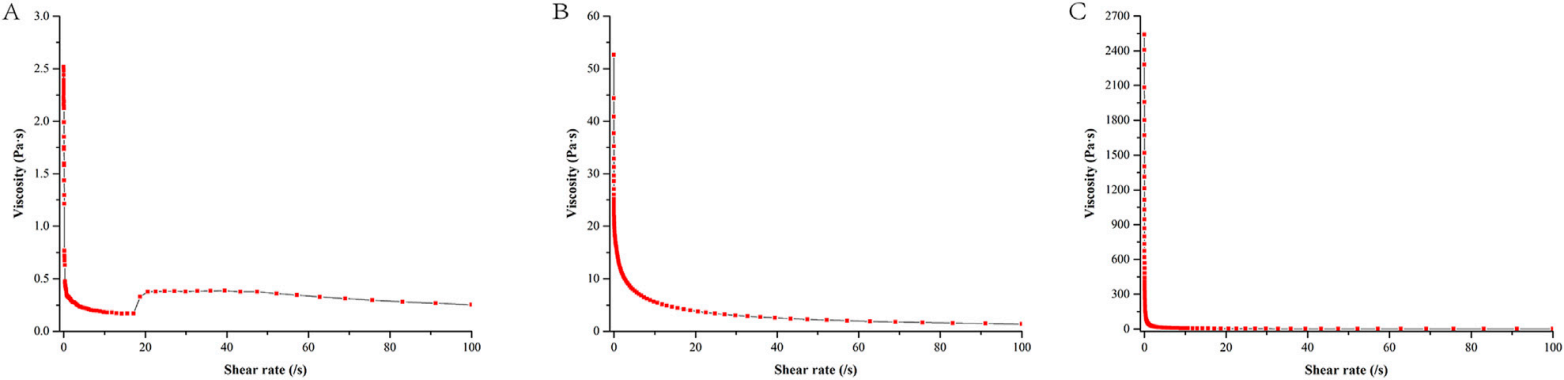
Shear rate-viscosity curve of different NE gel formulations. Formulation **(A)** utilizes HPMC, formulation **(B)** uses CMC-Na, and formulation **(C)** employs CP940.

Figure 4 illustrated that when CP940 is employed as the gel material, the initial viscosity of the NE gel (at a shear rate of 0) is measured to be 2,407.7 Pa•s, which is markedly higher than the other two gel formulations. As the shear rate increases, there is a notable decline in viscosity, exhibiting a distinct shear-thinning behavior. In contrast, when CMC-Na and HPMC serve as the gel matrices, the initial viscosity is comparatively lower, and the viscosity diminishes at a moderate pace as the shear rate intensifies.

**FIGURE 5:**
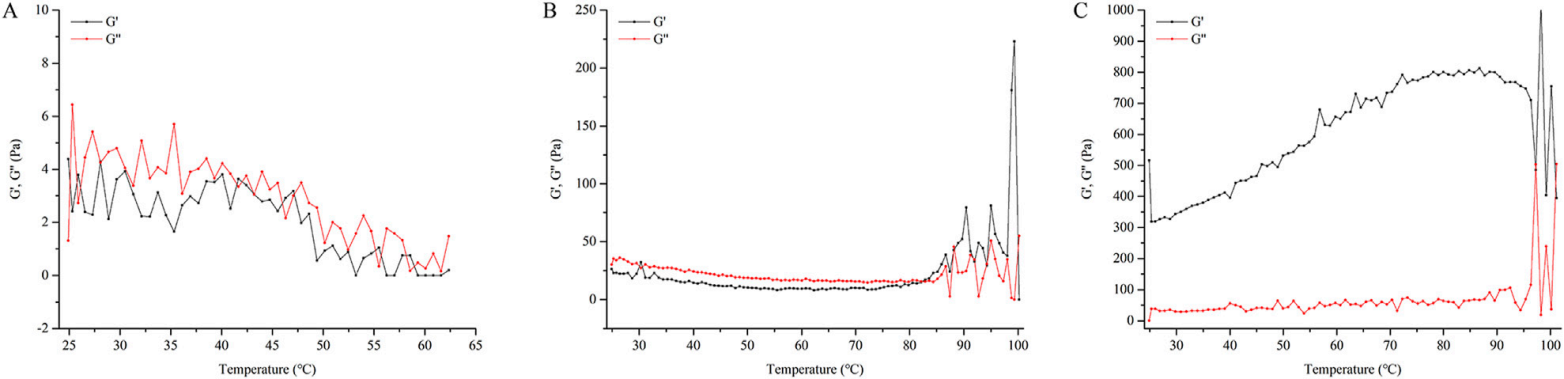
The relationship between the G′, G″ with temperature for different NE gel formulations. Formulation **(A)** utilizes HPMC, formulation **(B)** uses CMC-Na, and formulation **(C)** employs CP940.

As observed in Figure 5, the G′ of HPMC and CMC-Na are much lower than that of CP940, while the G″ of HPMC and CMC-Na are also much lower than that of CP940. Furthermore, for CP940, G′ > G″, in contrast, the NE gels based on HPMC and CMC-Na have G′ values close to their G″ values, suggesting they behave more like fluids. Consequently, CP940 was chosen as the gel material for further study.

### 3.4 *In vitro* skin permeation and drug retention assay

**FIGURE 6:**
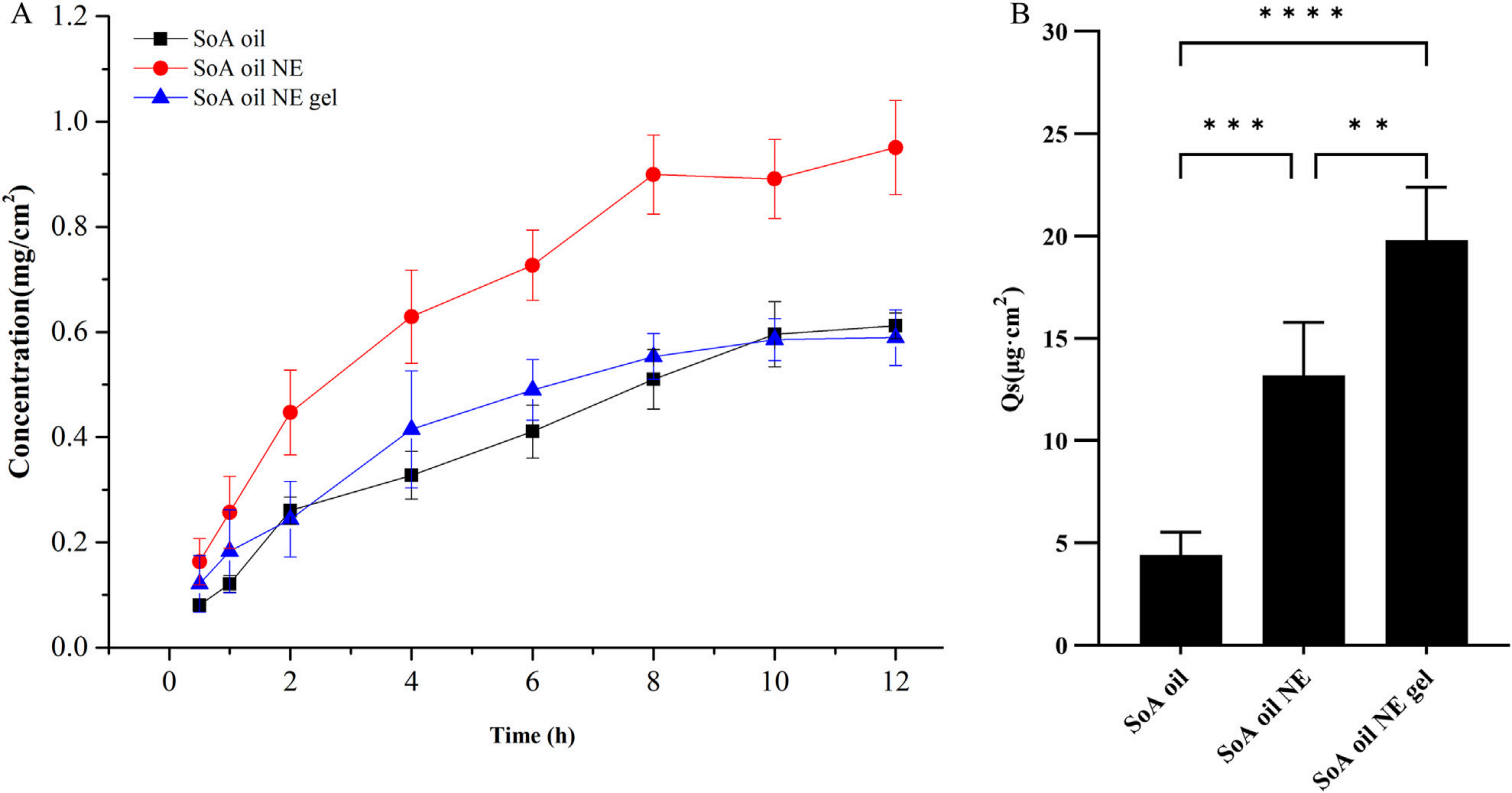
*In vitro* percutaneous permeation of oxymatrine through fresh rat skin at 37°C ± 0.5°C (n = 5), **(A, B)** are for skin penetration and retention of oxymatrine. **P < 0.01, ***P < 0.001, ****P < 0.0001 when compared with SoA oil samples.

The amount of oxymatrine permeated through the rat skin in a constant area within 12 h is shown in Figure 6A. Oxymatrine in the NE showed a higher amount of skin permeation than that in SoA oil. The amount of oxymatrine in the NE and the gel permeated through the skin in the initial 0.5 h was found to be 0.163 and 0.121 mg/cm^2^, which is 2-fold and 1.5-fold higher than SoA oil (0.081 mg/cm^2^), respectively. The NE enabled drug permeation at an amount of 0.951 mg/cm^2^ after 12 h, which is significantly higher than the SoA oil samples (0.612 mg/cm^2^), however the gel did not exhibit higher skin penetration properties than SoA oil (*P* > 0.05).

As depicted in Figure 6B, the drug retention of oxymatrine in skin samples following 12 h for SoA oil, the NE, and the gel was determined to be 4.4, 13.2, and 19.8 μg/cm^2^, respectively. The drug retention in both the gel and the NE samples is significantly higher compared to the SoA oil sample (*P* < 0.001, *P* < 0.0001). Moreover, there is a notable difference between the gel and the NE samples (*P* < 0.01), indicating that the gel exhibits superior skin retention properties over the other two formulations.

### 3.5 Skin irritation test

**FIGURE 7:**
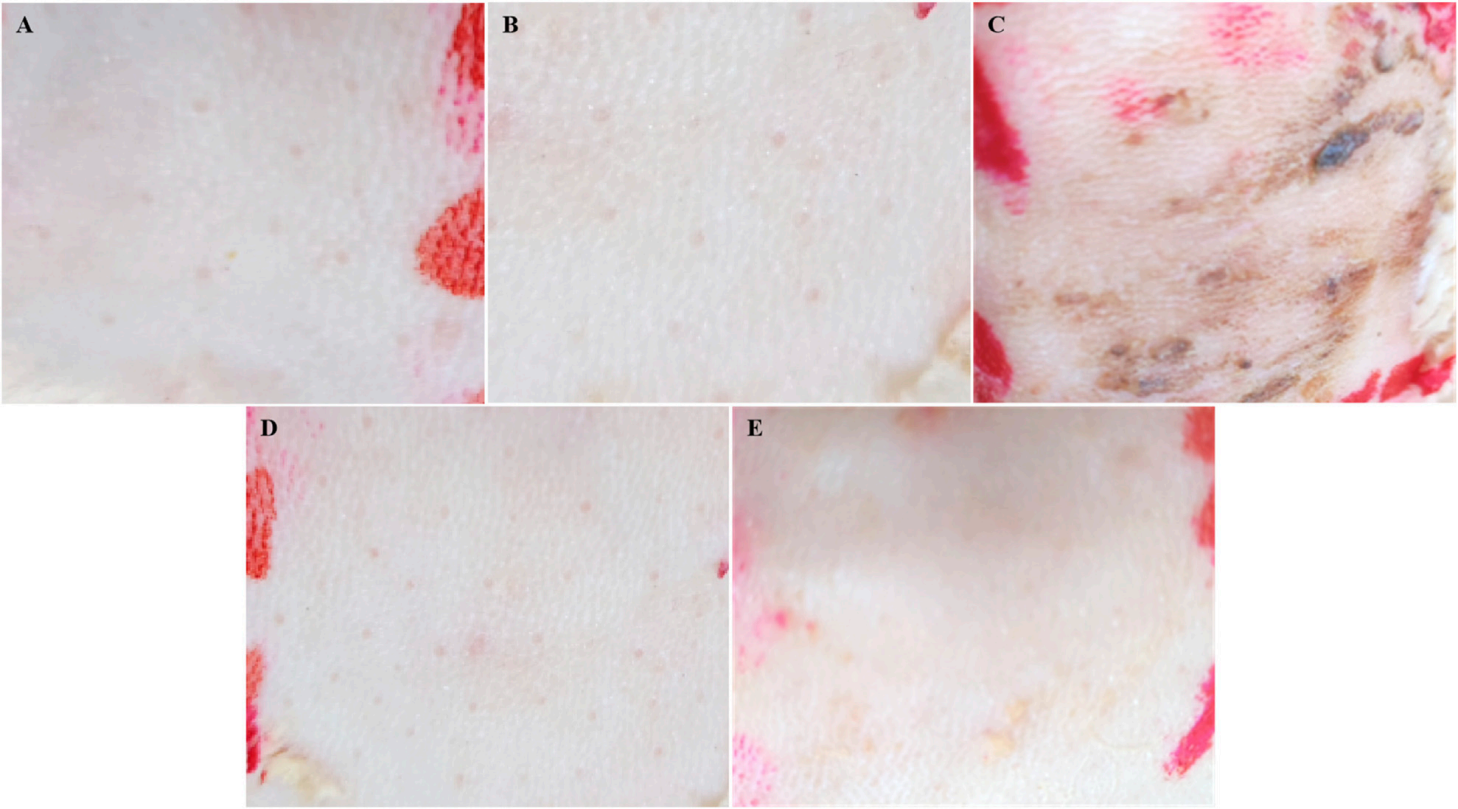
Skin irritation test was conducted on the dorsal skin of rabbits. **(A)** is for saline, **(B)** is for control group, **(C)** is for SoA oil, **(D)** is for the NE, and **(E)** is for the gel.

As illustrated in Table 1 and Figure 7, the dorsal skin of rabbits treated with SoA oil exhibited erythema and edema after 24 h, scoring 1 according to the irritation scoring system. As time progressed, the erythema and edema worsened, with the irritation score increasing to three at 48 h and further escalating to four at 72 h. No significant improvement was observed throughout the remainder of the experimental period, which concluded at 72 h. These findings suggest that SoA oil possesses moderate irritant properties towards rabbit skin. In contrast, the skin of rabbits treated with the NE, the gel, or the control group displayed no signs of erythema or edema, indicating a lack of overt dermal irritation.

**TABLE 1:** Skin irritation scores on rabbits dorsal skin.

Samples	Scores			
	1 h	24 h	48 h	72 h
Saline	0	0	0	0
Control	0	0	0	0
SoA oil	0	1	3	4
SoA oil NE	0	0	0	0
SoA oil NE gel	0	0	0	0

### 3.6 Biofilm inhibition efficacy

**FIGURE 8:**
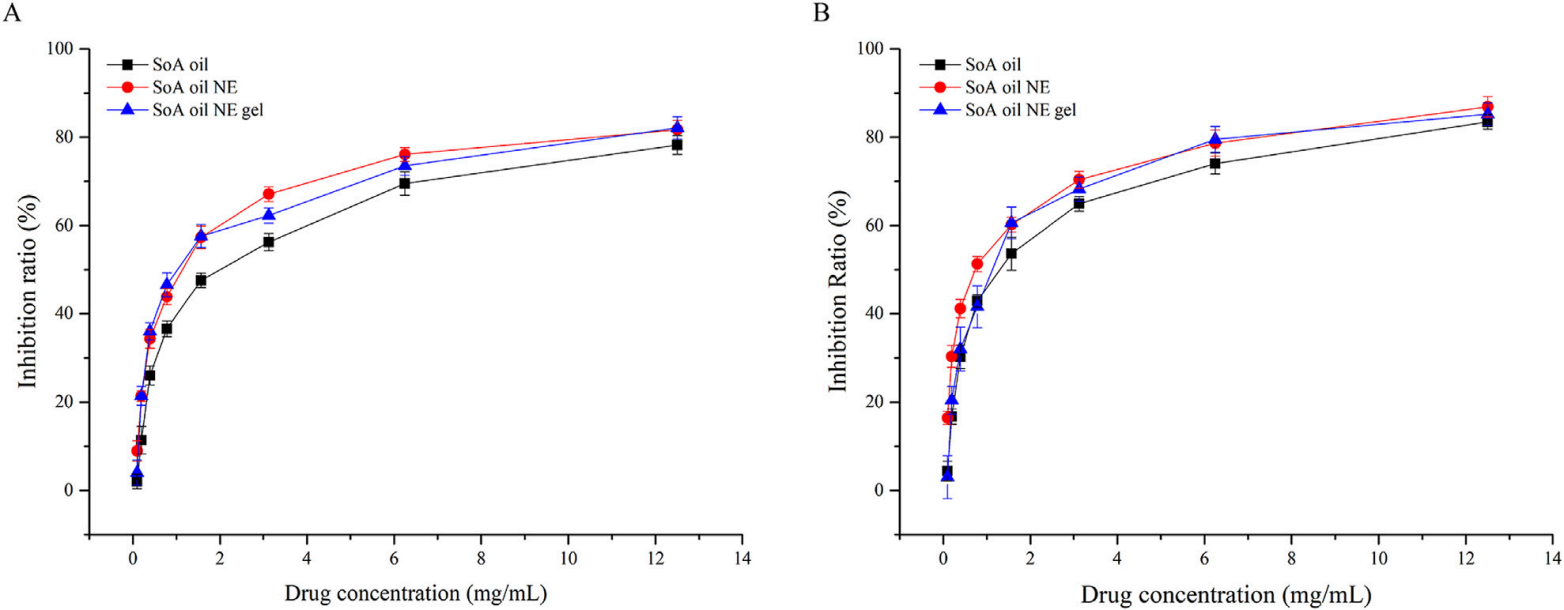
Inhibition rates of biofilm activity of SoA oil, the NE, and the gel against *S. aureus* **(A)** and MRSA **(B)**.

As illustrated in Figure 8A, the NE demonstrated superior biofilm inhibition against *S. aureus* at a low concentration (0.09766 mg/mL), achieving an inhibition rate of 8.9%, significantly outperforming SoA oil (2.0%) and the gel (4.0%). Dose-dependent manner of inhibition was observed across all formulations. At the concentration of 12.50 mg/mL, inhibition rates reached 78.3% (SoA oil), 81.7% (the NE), and 82.1% (the gel). While both NE and NE gel exhibited marginally stronger inhibition than unmodified SoA oil, statistical significance was not achieved (*P* > 0.05). Similarly, against MRSA biofilms (Figure 8B), all formulations displayed concentration-dependent inhibition. At 6.25 mg/mL, all formulations achieved approximately 80% inhibition, with a plateau effect observed at 12.50 mg/mL. Notably, within the range from 0.09766 to 3.12 mg/mL, the NE exhibited markedly higher anti-biofilm activity against MRSA compared to SoA oil and NE gel (*P* < 0.001).

### 3.7 Mature biofilm clearance efficacy

**FIGURE 9:**
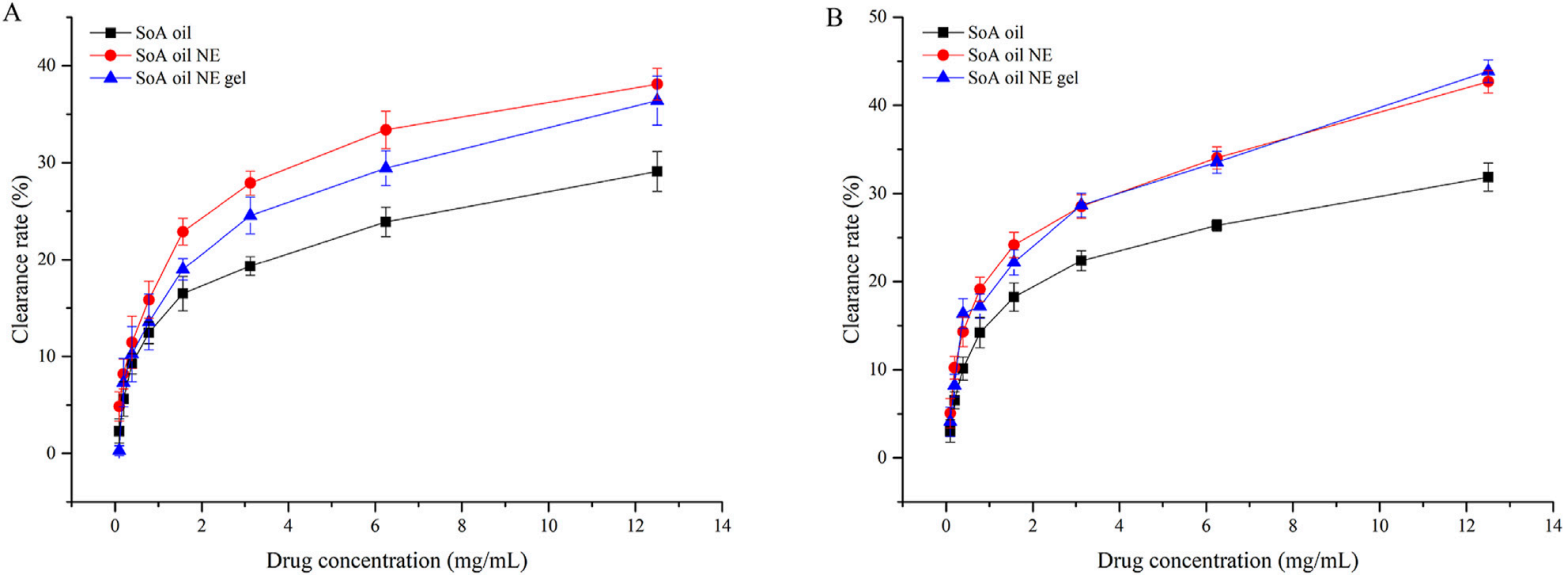
Clearance rates of mature biofilm activity of SoA oil, the NE, and the gel against *S. aureus* **(A)** and MRSA **(B)**.

For preformed *S. aureus* biofilms (Figure 9A), the NE achieved a 4.9% clearance rate at 0.09766 mg/mL, surpassing both SoA oil (2.3%) and NE gel (0.8%). Clearance rates increased dose-dependently, reaching 29.1% (SoA oil), 38.1% (NE), and 36.4% (NE gel) at 12.50 mg/mL. Both NE and NE gel exhibited statistically superior clearance efficacy relative to unmodified SoA oil (*P* < 0.0001). A parallel trend was observed against mature MRSA biofilms (Figure 9B). At 12.50 mg/mL, clearance rates were 31.9% (SoA oil), 42.7% (NE), and 43.9% (NE gel), with NE and NE gel demonstrating significantly enhanced activity compared to SoA oil (*P* < 0.0001).

## 4 Discussion

SoA has been extensively utilized in clinical settings due to its diverse pharmacological properties. However, current research has not specifically addressed its dosage form with the intent to enhance its poor transdermal permeability or to mitigate its irritancy. The percutaneous permeation of drugs is restricted by various factors, such as the barrier function of the stratum corneum (Bouwstra et al., 2023; Baveloni et al., 2021), the hydrophilic/hydrophobic properties of the drug (Wang et al., 2024; Jain et al., 2022), the dosage form of the drug (Supe and Takudage, 2021; Phatale et al., 2022), and the blood flow in the skin (Marwah et al., 2016), among which the barrier of the stratum corneum plays a crucial role in drug absorption.

NE have emerged as a focal point in pharmaceutical research due to their aptness for delivering both hydrophilic and lipophilic medications (Day et al., 2020; Poomanee et al., 2020). Comprising a blend of water, oil, and an mixed surfactant (Iskandar et al., 2024), NE exist as a thermodynamically stable and optically clear dispersion (Harimurti et al., 2021; Fryd and Mason, 2012). Notable attributes such as enhanced drug permeability (Moghassemi et al., 2022), and superior thermodynamic stability differentiate them from alternative carrier systems (Latif et al., 2022). Owing to these characteristics, NE are increasingly favored for use in transdermal drug delivery applications (Elsayed et al., 2024). In the present study, the optimized NE formulation exhibited a mean droplet size of 53.27 nm, which is smaller than 100 nm, and a PDI of 0.236, which is less than 0.5, suggesting a narrow size distribution (Alkilani et al., 2022; Al-Maqtari et al., 2021). The diminutive size coupled with the uniform dispersion of the NE underscores its exceptional stability (Alharbi et al., 2023). The failure of certain surfactant-to-cosurfactant ratios to form NEs can be attributed to the intricate balance required between the surfactant and cosurfactant components. This balance is crucial for reducing interfacial tension, stabilizing the oil-water interface, and ensuring the formation of fine, stable droplets. The hydrophilic-lipophilic balance (HLB) of the surfactant and cosurfactant mixture plays a significant role in this process (Magrode, et al., 2024; Smail et al., 2021).

The gel, compared to SoA oil alone, does not significantly enhance the percutaneous penetration of the drug but markedly increases the retention of the drug within the skin. In the present study, the NE gel derived from CP940 exhibits a high viscosity, and moreover, the G′ was larger than G″, reflecting its superior elasticity and stability (Zhao et al., 2020), which allows it to withstand deformation more effectively under external forces (Sheikh et al., 2023). Thixotropy is a rheological property where the viscosity of a material decreases under shear stress but recovers over time when the stress is removed. This behavior is particularly relevant for gels, as it affects their flow properties and stability. In our study, we observed that the gel exhibited thixotropic behavior, which is essential for its application. This property allows the gel to flow easily when applied but regain its structural integrity once the shear stress is removed, ensuring that it remains in place on the skin (Hashemnejad et al., 2019). Gels can enhance skin permeability through various mechanisms, including alteration of the stratum corneum lipid structure (Pan et al., 2022), and extraction or fluidization of stratum corneum lipids (Lupi et al., 2016). Furthermore, with good biocompatibility and appropriate viscosity (Khurana et al., 2013), gels can adhere closely to the skin surface, forming a continuous drug delivery layer. This intimate contact increases the duration of drug action, raising the likelihood of penetration.

After a course of consecutive 7 days of application, both the NE and the gel formulation demonstrated non-irritating properties when applied to the skin of rabbits. In contrast, the SoA oil induced the formation of erythema and edema, manifesting as moderate skin irritation. The small droplet size in NE provides a large surface area for drug release (Freytag and Odermatt, 2016), gels enabling controlled and sustained delivery, which can decrease the concentration of drug exposed to the skin at any given time (Baveloni et al., 2021; Kobra et al., 2023). The gel exhibited a elevated water content and permeability, coupled with negligible skin irritation, thereby potentially enhancing patient adherence to the therapeutic regimen (Agrawal et al., 2017).

The emergence of multidrug-resistant pathogens, particularly biofilm-forming strains like MRSA, necessitates innovative strategies to combat biofilm-associated infections. Our findings demonstrate that the NE and the gel formulation exhibit significant biofilm inhibition and clearance activities against both *S. aureus* and MRSA. NEs are known for their ability to enhance the penetration and distribution of active ingredients within biofilms. The small droplet size and high surface area of NEs facilitate better diffusion through the biofilm matrix, allowing the active components of SoA oil to reach deeper layers of the biofilm where bacteria reside. This improved penetration is crucial for effective eradication of biofilms, as conventional antibiotics often fail to penetrate beyond the surface layers due to the protective exopolysaccharide (EPS) matrix (Guo et al., 2017). Furthermore, the NE formulations can disrupt the biofilm matrix, making it more permeable to the active ingredients. This disruption is likely due to the surfactant properties of the NE components, which can interact with the EPS matrix and weaken its structure. This effect is further supported by studies showing that NEs can enhance the efficacy of antibiotics against biofilms by disrupting the protective barrier and increasing drug accessibility to bacterial cells (Papadopoulou, et al., 2023).

The antibacterial properties of alkaloids in *S. alopecuroides L.* are largely due to their ability to disrupt microbial cell membranes, inhibit enzyme activity, and impede the synthesis of critical bio-molecules such as proteins, RNA, and DNA within target microorganisms. Moreover, some alkaloids (matrine, oxymatrine, etc.) can effectively combat microorganisms that are resistant to traditional antibacterial agents by enhancing immune function and preventing biofilm formation (Thawabteh et al., 2021; Bhambhani et al., 2021; Heinrich et al., 2021; Plazas et al., 2022). The dose-dependent efficacy of these formulations aligns with previous studies highlighting the potential of plant-derived compounds to disrupt biofilm integrity through mechanisms such as quorum sensing interference, extracellular polymeric substance degradation, and membrane destabilization (Wang et al., 2015). Cannabinoids from Cannabis sativa reduce MRSA biofilm biomass by 71% at sub-MIC concentrations, likely through membrane disruption and altered metabolic activity. These observations underscore the multifaceted action of natural compounds in targeting biofilm resilience (Valliammai et al., 2019; Roshan et al., 2024). The superior performance of the NE compared to unmodified SoA oil may stem from its enhanced bioavailability, a trait shared with nanotechnology-based delivery systems. For example, liposomal polymyxin B improve biofilm penetration and sustained antimicrobial activity (Fu et al., 2019).

## 5 Conclusion

In this study, a pseudo-ternary phase diagram was employed to optimize the surfactant and co-surfactant composition for the NE, resulting in a formulation with minimized particle size and uniform dispersion. This optimized NE was further incorporated into a gel formulation. The rheological properties of the resultant gel were comprehensively evaluated.

The integration of SoA oil into a nanoemulsion-based gel significantly increased the drug’s cutaneous retention while mitigating skin irritation. These findings provide a solid foundation for the future development and clinical application of SoA oil formulations. Moreover, this study underscores the potential of plant-derived nanoformulations and surfactants as viable alternatives to conventional antibiotics for treating biofilm-associated infections.

However, this study has certain limitations. Specifically, the stability of the NE and the gel formulation was not evaluated. Though our *in vitro* studies have demonstrated promising results in inhibiting biofilm formation, we acknowledge that these findings need to be further validated in animal models to better understand the gel’s efficacy and safety in a more complex biological environment. In addition, the *in vivo* pharmacodynamics and mechanisms of action were not thoroughly explored. Future research will focus on addressing these limitations to further elucidate the potential of SoA oil nanoformulations for clinical use.

## Data Availability

The original contributions presented in the study are included in the article/supplementary material, further inquiries can be directed to the corresponding author.
